# Doppler radar rainfall prediction and gauge data

**DOI:** 10.1186/s13104-020-05311-y

**Published:** 2020-10-14

**Authors:** Jesse W. Lansford, Tyson H. Walsh, T. V. Hromadka, P. Rao

**Affiliations:** 1 Department of Mathematics, United States Military Academy, West Point, NY USA; 2 Department of Civil and Environmental Engineering, California State University, Fullerton, CA USA

**Keywords:** Hydrometeorology, Engineering, Floodplain management, Weather forecasting

## Abstract

**Objective:**

The data herein represents multiple gauge sets and multiple radar sites of like-type Doppler data sets combined to produce populations of ordered pairs. Publications spanning decades yet specific to Doppler radar sites contain graphs of data pairs of Doppler radar precipitation estimates versus rain gauge precipitation readings.

**Data description:**

Taken from multiple sources, the data set represents several radar sites and rain gauge sites combined for 8830 data points. The data is relevant in various applications of hydrometeorology and engineering as well as weather forecasting. Further, the importance of accuracy in radar and precipitation estimates continues to increase, necessitating the incorporation of as much data as possible.

## Objective

Prediction of rainfall using Doppler technology relies on synthesizing the signal information that the radars receive back from the atmosphere. Processing this signal information yields a rainfall estimate relying on relationships between statistical regression equations and several other parameters. Herein we present the ordered data for both Doppler radar precipitation estimates with associated gauge precipitation estimates pulled from ten referenced publications. Researchers may use these rainfall estimates for study purposes by comparing the accuracy of the Doppler radar estimated precipitation with the actual comparative gauge data. An indication of the estimation error can be determined by comparing the frequency distribution of the source data against the regression equation predictors obtained from the Doppler radar estimates.

This data is relevant to other researchers involved in investigating the association of Doppler radar data and gauge precipitation estimate relationships [1].

## Data description

Datasets generated and/or analyzed during the current study are available in the figshare repository: 10.6084/m9.figshare.11811675.v1 [2].

Published literature contains data in the form of scatter plots and tables [3–12]. The data compares Doppler radar derived rainfall estimates with observed local gauge values, spread across storms and geographical domains with the majority categorized via total storm accumulation. Pulling from each reference, digitizing software reads graphs and tabulates data for later concatenating. Specifically, the plot digitizer (http://plotdigitizer.sourceforge.net/), is a Java tool that digitizes data points from scanned plots. Note that the data used for analysis is *not* raw data; it is the end data obtained by the respective authors after applying standard corrections (if any) to the radar reflectivity and other parameters. The gauge data are also the final data as recorded by the gauges at the respective locations. There is no need for additional corrections or processing for the published data in the cited references. The data used in the referenced analysis [1] are from the original, cited manuscripts. Assume that the displayed data points relating to the correlation between Doppler-Radar readings versus measured precipitation, as shown in the various figures of the published papers shown in the references, are adequately processed and suitable for further analysis. Without the actual source data, the processed data supplied by the respective papers’ authors or digitizing the data directly from the papers used, populated the ordered pairs in the presented data set. Assume that standard procedures for processing the source data were used for the various papers assembled from the literature. These standard procedures as detailed by the respective authors relate to the geographical location of radar and gauge. Similarly, the rain-gauge data are obtained from the papers’ authors or by digitizing the point data displayed in the respective paper’s diagrams and figures.

The data file (Table 1) comprises two columns of rainfall data; Gauge and Radar. Gauge consists of rainfall values in millimeters given by the recording gauge following the precipitation event. Radar consists of estimated rainfall values in millimeters given by the Doppler radar estimates prior to the precipitation event. Concatenating these two columns results in 8830 ordered pairs.

**Table 1:** Overview of data files/data sets

Label	Name of data file/data set	File types (file extension)	Data repository and identifier (DOI or accession number)
Data file 1	DopplerWeatherData	Comma Separated Values (.csv)	10.6084/m9.figshare.11811675.v1 [2]

We did not seek other information such as measurement site location and collection time. This paper attempts to develop a correlation between published, open literature versus possible uncertainty in using such information. Further, we did not seek underpinnings of how published correlations between measured information and gage data were formulated. Rather, we focus on the data as published and what generalizations can be made about Doppler-Radar and Dual Doppler-Radar versus uncertainty in predicting measured precipitation on the ground. Although true that an indication of estimation error can be determined by comparing the frequency distribution of the source data against the regression equation predictors obtained from the Doppler radar estimates, this research effort does not move towards resolving such factors in order to be able to assess the value of direct use of published information.

## Limitations

Due to the replacement of Doppler radars with dual-polarization radars, there is currently no more data being collected for additional analysis.

## Data Availability

The datasets generated during and/or analysed during the current study are available in the figshare repository: 10.6084/m9.figshare.11811675.v1 [2].
